# Pros and Cons of the SeHCAT Test in Bile Acid Diarrhea: A More Appropriate Use of an Old Nuclear Medicine Technique

**DOI:** 10.1155/2018/2097359

**Published:** 2018-11-26

**Authors:** Bernardo Fani, Lorenzo Bertani, Italia Paglianiti, Lorenzo Fantechi, Nicola De Bortoli, Francesco Costa, Duccio Volterrani, Santino Marchi, Massimo Bellini

**Affiliations:** ^1^Department of New Technologies and Translational Research in Medicine and Surgery, Gastroenterology Unit, University of Pisa, Via Roma 67, 56122 Pisa, Italy; ^2^Department of New Technologies and Translational Research in Medicine and Surgery, Nuclear Medicine Unit, University of Pisa, Via Roma 67, 56122 Pisa, Italy; ^3^Department of General Surgery and Gastroenterology, Gastroenterology Unit, Az. Ospedaliero Universitaria Pisana, Via Paradisa 2, 56124 Pisa, Italy

## Abstract

Bile acid malabsorption (BAM) causing chronic diarrhea may be due to organic as well as functional disorders, and some of them were included under the general label of diarrheic-type irritable bowel syndrome (IBS-D). The 75-selenium homocholic acid taurine (SeHCAT) test is a nuclear medicine investigation considered to be the gold standard for the diagnosis of bile acid malabsorption (BAM). Many studies demonstrate that it could be effective in the clinical workout of chronic diarrhea due to different conditions. The SeHCAT test provides a quantitative assessment to estimate the severity of BAM and the possible response to therapy with bile acid sequestrants (BASs). However, there is no general agreement regarding its cutoff value and the test is not widely available. The aim of this review is to discuss the advantages and disadvantages of the SeHCAT test in clinical practice.

## 1. Introduction

Bile acid diarrhea (BAD) is caused by bile acid malabsorption (BAM). It is characterised by watery diarrhea (often postprandial), bloating, urge for defecation, and faecal incontinence at times.

**Figure 1:**
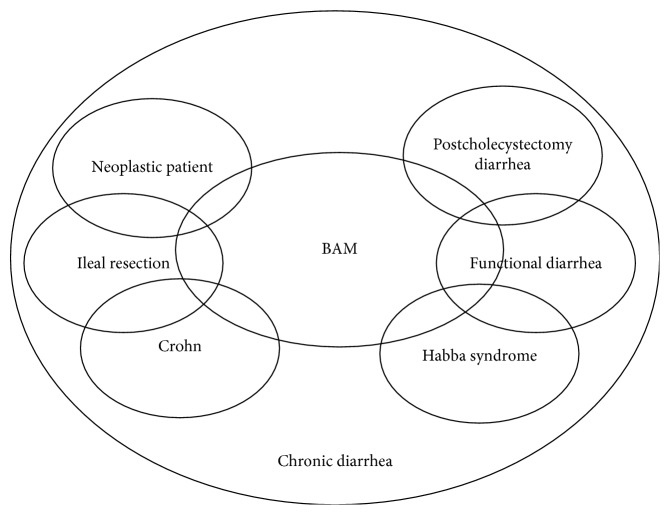
Venn diagram of different causes of chronic diarrhea related to BAM.

BAM may occur in many different pathological conditions, which sometimes overlap (Figure 1). It is estimated that about 1% of the general population is affected by BAD [1]. BAM is often caused by a surgical resection or a structural impairment of the ileum (i.e., Crohn's disease (CD)), but many studies [2] demonstrate the presence of BAM in patients with predominant irritable bowel syndrome diarrhea (IBS-D) or with functional diarrhea (FD).

Clinically, BAM is classified as follows [3]: type 1: ileal dysfunction/resection (Crohn's disease); type 2: primary or idiopathic, characterised by watery diarrhea with (IBS) or without (FD) pain responding to bile acid sequestrant drugs (BASs); type 3: associated with other gastrointestinal disorders such as coeliac disease, small intestinal bacterial overgrowth (SIBO), and chronic pancreatitis; and type 4: due to an impaired FGF-19 feedback inhibition that causes excessive BA synthesis [4].

Currently available therapies are BASs like cholestyramine, colestipol, and colesevelam. Obeticholic acid could be a promising drug for BAM: it is an agonist of the farnesoid X receptor (FXR), which increases fibroblastic growth factor 19 (FGF-19) synthesis and decreases bile acid (BA) synthesis by hepatocytes [3, 5].

## 2. Pathophysiology

**Figure 2:**
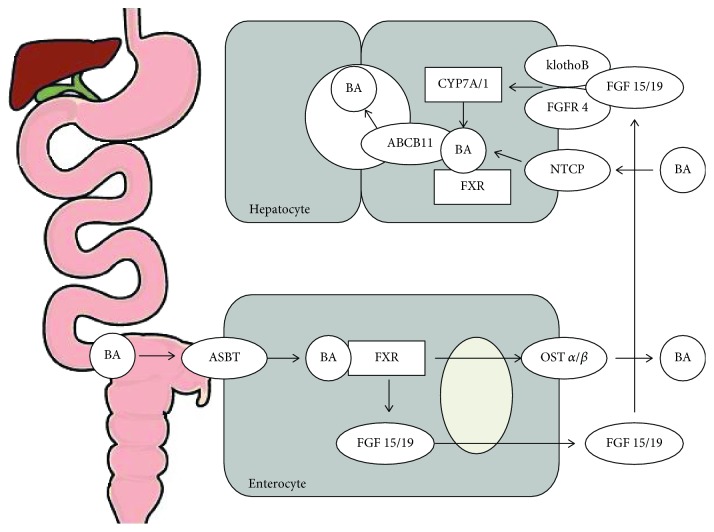
Pathophysiology of enterohepatic circulation: bile acids (BAs) excreted in the intestinal lumen are mainly reabsorbed in the ileum through the apical sodium-dependent bile acid transporter (ASBT) and return to the liver through the portal vessels. Stimulation of the farnesoid X receptor (FXR) initiates the production of fibroblastic growth factor 15/19 (FGF-15/19) that interacts in the hepatocytes with cholesterol 7 alpha-hydroxylase (CYP7A/1) and reduces BA synthesis, with a negative feedback mechanism. Mutations in ASBT and klothoB have been demonstrated to be a cause of bile acid malabsorption (BAM).

BAs excreted into the intestinal lumen are mainly reabsorbed in the ileum by a specific receptor, the apical sodium-dependent bile acid transporter (ASBT), and return to the liver via the portal venous system (Figure 2).

BAs entering the enterohepatic circulation are primary acids synthesised from cholesterol in the hepatocytes. They are actively secreted across the canalicular membrane and carried into the bile to the gallbladder, where they are concentrated in the fasting state. In a single pass of clearance, about 95% of BAs are actively absorbed from the lumen of the terminal ileum, leaving only approximately 5% in the colon, where a fraction is passively reabsorbed after some modifications including deconjugation and oxidation of hydroxyl groups [6]; hydrolysis and hydroxyl group dehydrogenation reactions are performed by a broad spectrum of intestinal bacteria [7].

BAM can occur due to genetic mutations of the ASBT receptor, which is the ileal transporter involved in the reabsorption of BAs [8]. Also, genetic mutations of TGR5, a G-protein-coupled receptor that functions as a cell surface receptor for BAs and regulates basal and cholinergic-induced secretion in the colon and colonic transit, can predispose to BAM [9]. Another important cause of BAM may be a deficiency in FGF-19, a hormone produced in the enterocytes that regulates hepatic BA synthesis via a negative feedback mechanism [4, 10]. Walters et al. reported lower serum FGF-19 in patients with BAM and an inverse relationship between FGF-19 and serum C4 (a surrogate index of hepatic BA synthesis) [4]. Genetic variations in the proteins involved in the feedback regulation of BA synthesis, specifically the klothoB gene and fibroblast growth factor 4 gene (Figure 2), are rare causes of BAM. A significant association of SNP rs17618244 in the klothoB (KLB) gene with colonic transit in IBS-D has been demonstrated [11]. An accelerated small-bowel transit, bypassing active BA transport in the ileum, has been hypothesised as a cause of BAM in idiopathic [12] and postradiation cases [13].

## 3. Diagnosis

The diagnosis of BAM can be obtained using (1) 14C-glycocholate breath and stool test, (2) determination of serum C4 (7*α*-hydroxy-4 cholesten-3-one) or FGF-19 levels, (3) 24-hour faecal BA output dosage, and (4) 75-selenium homocholic acid taurine (SeHCAT) test.

The 14C-glycocholate breath and stool test has a limited clinical use because it is cumbersome and time-consuming [14, 15].

Determination of serum C4 levels using a relatively simple chromatographic method is potentially applicable to most patients, but it requires further clinical validation. It varies according to a circadian rhythm, and false-positive results are reported in patients with liver disease or in those treated with statins. The method has a specificity and sensitivity of 90% and 77%, respectively, for BAM type 1 and 97% and 74%, respectively, for BAM type 2 [16].

Serum FGF-19 levels measured using ELISA are inversely related to C4. Recent data show that FD and IBS-D are associated with increased serum C4 levels and total faecal BAs and with decreased serum FGF-19 levels [17].

The assessment of the 24-hour faecal output of BAs is cumbersome and not widely available. An enzymatic assay indirectly measures faecal BAs, but it tends to underestimate total BAs [18]. Moreover, when it is used to measure BA concentrations in small-bowel fluid or in ileostomy effluent, it is not reliable [19].

A possible diagnostic option could be an empiric trial with BASs. Cholestyramine is given for 10 days with a variable dosage (4–36 g/day) [20]. In patients with symptom improvement, the treatment may be stopped and, if the BAM symptoms reappear after seven days, the test is considered positive. BAM diagnosis with a cholestyramine trial is less expensive and immediately available. Unfortunately, this is not supported by any quantitative data but only by the presence or the absence of a clinical improvement referred by the patients. Moreover, evaluating the clinical response to BASs may give false positive for a placebo effect or false negative for poor compliance with the therapy [21]. Furthermore, the lack of specificity—since cholestyramine may inactivate some diarrhea etiological agents such as the *Clostridium difficile* toxin [22, 23], the possible adverse events associated with BASs (e.g., drug interactions), and the difficulty in determining the effective dosage are not to be neglected.

## 4. The SeHCAT Test

The SeHCAT test, performed at the Nuclear Medicine Department, is simple, fast, and well tolerated and requires two scans one week apart. The SeHCAT test is the gold standard for diagnosing BAM [24] because of its highest sensitivity and specificity [25]; Merrick et al. [26] demonstrated a sensitivity of 100% and a specificity of 91% with a cutoff value of 15%.

This test was first performed in 1981 [27], and the protocol currently used was developed by Brydon et al. [16]. The SeHCAT test is currently available in twelve European countries and in Canada, but not in the USA; it is relatively expensive and it is usually available only at third-level centers. The SeHCAT test measures the whole body retention of a radiolabelled taurine-conjugated bile acid analogue (^75^Se) after seven days; a retention value of ≤10–15% is usually considered diagnostic [1].

The standard patient preparation requires the suspension of bile acid sequestrants and antidiarrheal drugs one week prior to the first appointment because of interference with test results and requires fasting at least 4 hr before taking the SeHCAT capsule (GE Healthcare).

The administered SeHCAT activity is very small (370 kBq), with an effective dose of radiation for an adult of 0.26 mSv and total absorbed radiation of 0.3 Gy/kBq. The absorbed dose for the small intestine and gallbladder is 3.0 and 3.2 Gy/kBq, respectively [28]. The absorbed dose for the small bowel increases in patients who have undergone cholecystectomy and who have severe liver damage. In comparison, the radiation dose given during an abdominal CT scan is approximately 5.3 mSv and the background annual exposure in the UK is 1–3 mSv [29].

The first scan is usually performed 3 hr after SeHCAT capsule ingestion (370 kBq) while patients are still fasting. The second scan is scheduled 7 days after capsule administration, which represents the optimal rescan time to calculate the SeHCAT % retention and to differentiate the normal biliary acid retention (95%) from abnormalities. The body's retention of the radiotracer correlates with ileal absorption [30].

**Figure 3:**
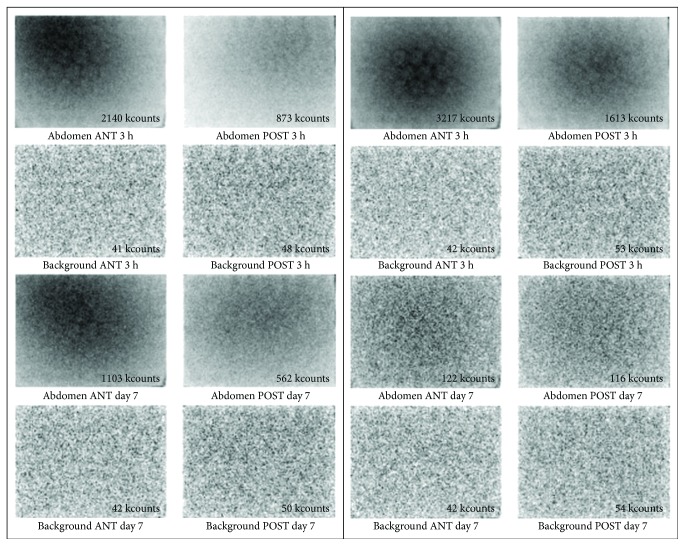
Examples of noncollimated abdomen images and related backgrounds at days 0 and 7 of a pathologic (a) and normal (b) SeHCAT test.

There are different gamma camera measurement methods—whole-body count and static abdomen acquisition with or without collimators (Figure 3). The total body or abdominal acquisition is performed with the detector of the gamma camera at the maximum opening of the gantry.

The abdominal acquisition is performed with patients in a supine position in an uncollimated gamma camera and by acquiring total counts in 5 min, preceded or followed by a background count acquisition either in the early (3 hr) or in the late (7 days) scan with equal duration and position. Care should be taken with abdominal acquisition, as it requires identical patient positioning inside the camera between the first and the second scan [31].

Collimation removal improves test sensitivity and accuracy, although, depending on the model of the gamma camera, it may be necessary to pay attention to possible crystal damage.

Abdominal counts are corrected for background (BG) counts and for decay; 75-selenium has a relatively long half-life (118 days). The percentage of abdominal (or whole-body) retention is calculated according to the following formula [32]:

(1) $$\begin{array}{c} \%\;retention\;at\;day\;7=\frac{1.04*\sqrt{\left(Ant\;Counts\;day\;7-Ant\;BG\;counts\right)}*\sqrt{\left(Post\;Counts\;day\;7-Post\;BG\;counts\right)}}{\sqrt{\left(Ant\;Counts\;day\;0-Ant\;BG\;counts\right)}*\sqrt{\left(Post\;Counts\;day\;0-Post\;BG\;counts\right)}}*100. \end{array}$$

**Figure 4:**
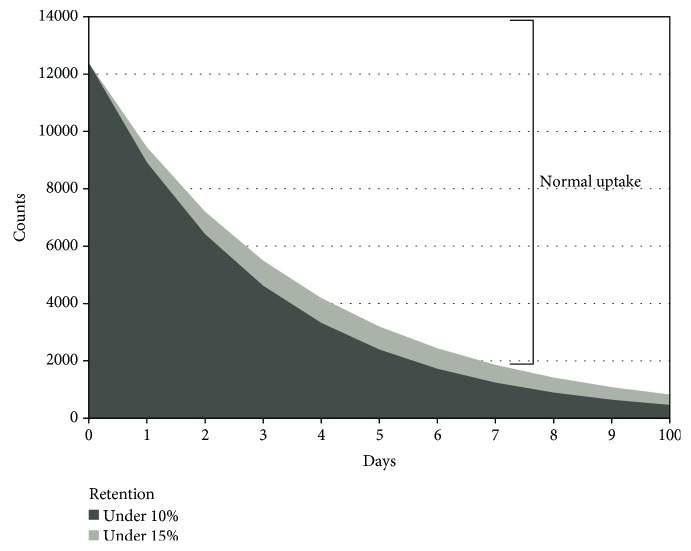
The lines show the threshold levels of the normal uptake value of SeHCAT from day 0 to day 10 evaluated for retention of 10% and 15% at day 7.

This method is used by many authors and gives a reliable measure of SeHCAT retention.  Figure 4 shows the 75-selenium retention in a graphical format, which includes a cutoff line for abnormality for days 0–10.

The optimal cutoff level to diagnose BAM varies from ≤10% to ≤15% [2]. Initially, a cutoff value of ≤15% was used, but later, in 1994, this value was revised to ≤10% [33]. Considering a theoretical abnormal reabsorption of bile acids of 94% in the enterohepatic circulation, on the seventh day, the estimated cutoff retention value would be 10%. Therefore, it seems reasonable to consider this the optimal cutoff level [34]. In addition, Wedlake et al. [1] observed that the response to cholestyramine was better using 10% than using 15% as the cutoff level (response rate 80% vs. 70%, respectively).

Many studies classify BAD into mild, moderate, and severe, on the basis of retention values on the seventh day of ≤15%, ≤10%, and ≤5%, respectively [26, 33, 35–39]. A recent review [25] shows the following percentages of response to BAS therapy: mild BAM—73%, moderate BAM—76%, and severe BAM—88%. BAM severity could allow the clinician to predict the response to therapy and is a starting point for evaluating clinical improvement.

Moreover, a positive diagnosis can have a positive psychological impact on the patient, leading to a better compliance with BAS therapy. Patients with a confirmed diagnosis are more motivated to start and continue a treatment with BASs, which are not palatable and can potentially induce some adverse events [40]. This is especially true for patients with FD or IBS-D who have often undergone many different diagnostic tests. The diagnosis of BAM in patients with chronic diarrhea has a great clinical relevance: since a positive SeHCAT test does not exclude other organic causes of diarrhea, patients should also undergo other tests as clinically indicated. This is particularly true in patients treated with many different drugs, where the cause of diarrhea may be difficult to diagnose. The use of the SeHCAT test in these instances may help in reaching the correct diagnosis. In an open multicenter study [41] conducted in 98 IBS-D patients who underwent the SeHCAT test, 56 patients showed altered SeHCAT retention and 42 completed a course of cholestyramine therapy at the mean dose of 4.8 g per day: only three did not respond to the drug.

SeHCAT test enables the clinician to make a more rational use of BASs which may induce adverse events. Prolonged BAS treatment may lead to malabsorption of fats and liposoluble vitamins (A, D, and K), increasing the risk of osteoporosis and possible coagulation abnormalities. For this reason, patients with coagulation defects or those taking oral anticoagulant therapy should undergo a SeHCAT test to obtain a precise diagnosis and to evaluate the benefit-risk ratio of BAS administration, as well as patients assuming life-saving drugs whose absorption could be potentially modified by BASs.

Despite the high percentage of patients with a positive SeHCAT test responding to BASs, there are currently limited data on the duration and the dosage of the BAS therapy and whether a clinical remission (negative SeHCAT test) after a long-term BAS treatment is possible [33, 42].

## 5. Use of the SeHCAT Test in Patients with Organic Diseases

In some cases, the SeHCAT test can provide important additional information.

### 5.1. Inflammatory Bowel Disease (IBD)

In CD with ileal involvement [43], BAM was diagnosed in 116/276 (42%) patients; as expected, the most severe BAM was observed in CD patients with more severe ileal involvement or after resection of the distal ileum. It is common to find CD patients with persistent diarrhea despite having normal inflammatory and disease activity indexes. BAM should be suspected as a cofactor of diarrhea as IBS may coexist in IBD patients [44]. A Dutch study highlighted that the majority of IBD patients with IBS-type symptoms fulfilled the criteria for IBS or mixed-type IBS [45]. Patients with CD and unexplained persistent diarrhea without disease activity should be screened for BAM [43] because therapeutic response to BASs is related to BAM severity [35, 39, 46].

### 5.2. Neoplastic Disease and Postsurgical Patients

Gastrointestinal symptoms are common consequences of many cancer treatments and have great impact on a patient's daily activities [47]. Diarrhea is one of the most frequent symptoms during chemotherapy or radiotherapy, and BAM could be involved in its pathophysiological mechanism [47].

In a study by Phillips et al. [13], 215 out of 506 (42.5%) consecutive neoplastic patients treated with surgery, radiotherapy, or chemotherapy and sent to a gastroenterological evaluation for diarrhea reported a new diagnosis of BAM with the SeHCAT test. It was mild in 25.6%, moderate in 29.3%, and severe in 45.1%. Since the adverse effects of BASs [37] (namely, interference with the absorption of micro-/micronutrients and drugs) may be particularly dangerous in these patients, a diagnosis of BAM should be carefully established before starting BAS therapy, which could significantly improve symptoms and quality of life. A SeHCAT test scan should be considered by the gastroenterologist treating cancer patients with diarrhea [13].

Up to 89% of the patients who have undergone Whipple's procedure may show a positive SeHCAT test [13]. In these patients, BAM could occur because of the associated vagotomy, which accelerates gut transit [48], but more probably it could be due to an alteration in bile production and/or an overrapid intestinal transit, leading to malabsorption of BAs. An interference from nonhydrolysed triglycerides, which also impairs absorption, like in chronic pancreatitis and pancreatic disease (i.e., cystic fibrosis), could also be possible [49, 50].

### 5.3. Cholecystectomy

BAM occurs in more than 90% of patients with postcholecystectomy diarrhea (PCD) [51]. The pathophysiological mechanism is linked to the lack of BA reservoir and the consequent inability of the gut to absorb their excessive output. A study by Sciarretta et al. [51] highlighted that patients with PCD responded favorably to cholestyramine (2–12 g/day) and, in 60% of the cases, they resolved their diarrhea after treatment withdrawal, despite persistent evidence of BAM. In fact, some studies have shown that BAS can improve diarrhea in many different pathological conditions, also in patients without BAM. In particular, cholestyramine, which is a strong anion-exchange resin that can bind with bacterial toxins and mycotoxins in the colon [46], was effective in improving diarrhea also in patients with microscopic colitis without associated BAM, as reported by Fernandez-Banares et al. [52]. It was also able to reduce the risk of developing *Clostridium difficile*-associated diarrhea due to its capacity of binding to toxins A and B [22]. This pharmacological effect could work in the cholecystectomized patient with a multifactorial diarrhea. This suggests the existence of other factors associated with BAM and, above all, a healing role rather than a symptomatic one for this drug.

### 5.4. Habba Syndrome

This syndrome is defined by the presence of abnormal gallbladder function and chronic postprandial diarrhea responding to BASs [53]. Hepatobiliary nuclear scintigraphy using Tc-99m-DISIDA with cholecystokinin (DISIDA with CCK injection) has to be performed to estimate the gallbladder ejection fraction, in accordance with the standard calculation of gallbladder contraction 30 minutes after CCK injection, to establish the possible relationship of gallbladder dysfunction and chronic diarrhea. An ejection fraction < 35% is considered grossly abnormal, 35–50% borderline abnormal, and >50% normal. The response of the diarrhea to BASs is probably due to a mechanism similar to that observed in PCD [51]. However, the poor function of the gallbladder seems to be the common primary factor in this syndrome. The SeHCAT test can provide further diagnostic confirmation, and data on the response to BAS therapy have already been discussed above.

## 6. Conclusions

The SeHCAT test is a safe and effective method to diagnose BAM with high sensitivity and specificity.  Table 1 summarizes the advantages and the disadvantages of the SeHCAT test.

Due to the high prevalence of BAM in FD and IBS-D, the SeHCAT test should be performed in chronic diarrhea with functional characteristics after evaluating FBC, CRP, coeliac serology, thyroid function, and stool exams (calprotectin, coproculture, ova, and parasites) [54, 55]. However, up to now, its cost and the lack of an agreed standard cutoff have strongly limited its wider acceptance and availability in everyday clinical practice. More widespread use of the SeHCAT test should provide further information to help understand the pathophysiologic mechanisms underlying chronic diarrhea afflicting many different patients and ensure that they are offered a treatment that is selected on the basis of a reliable clinical test and not on simple empirical observations.

## Figures and Tables

**Table 1:** Advantages and disadvantages of the SeHCAT test for BAM.

Advantages	Disadvantages
Gold standard for the diagnosis of BAM with high sensitivity and specificity	Relatively expensive and usually available only at third-level centers
Simple, safe, and well tolerated	SeHCAT does not exclude other causes of organic diarrhea
Quantitative evaluation of BAM predicts the response to therapy with BASs	The optimal cutoff level for diagnosis is not yet completely agreed by all centers
More rational use of BASs in relation to possible side effects	Diagnosis of BAM could be empirically obtained with a BAS trial
